# 2,2′-[Ethane-1,2-diylbis(­oxy)]dibenz­alde­hyde

**DOI:** 10.1107/S1600536813019156

**Published:** 2013-07-13

**Authors:** Mehmet Akkurt, Shaaban K. Mohamed, Peter N. Horton, Eman M. M. Abdel-Raheem, Mustafa R. Albayati

**Affiliations:** a Department of Physics, Faculty of Sciences, Erciyes University, 38039 Kayseri, Turkey; b Chemistry and Environmental Division, Manchester Metropolitan University, Manchester M1 5GD, England; c Chemistry Department, Faculty of Science, Mini University, 61519 El-Minia, Egypt; d School of Chemistry, University of Southampton, Highfield, Southampton SO17 1BJ, England; e Chemistry Department, Faculty of Science, Sohag University, 82524-Sohag, Egypt; f Kirkuk University, College of Science, Department of Chemistry, Kirkuk, Iraq

## Abstract

In the title compound, C_16_H_14_O_4_, the benzene rings are inclined at a dihedral angle of 75.14 (9)°. The torsion angle of the bridging O—C—C—O group is −76.50 (11)°. In the crystal, mol­ecules are linked by C—H⋯O hydrogen bonds, forming *C*(6) chains along [100]. Furthermore, C—H⋯π inter­actions and π–π stacking inter­actions [centroid–centroid distances = 3.6957 (7) and 3.6735 (8) Å] contribute to the stability of the crystal packing.

## Related literature

For the synthesis and utlization of bis-funtionalized compounds, see: Holland *et al.* (2007 ▶); Pedras *et al.* (2010 ▶); Mabkhot *et al.* (2012 ▶); Gavrilova & Bosnich (2004 ▶). For bond-length data, see: Allen *et al.* (1987 ▶). For graph-set theory, see: Bernstein *et al.* (1995 ▶).
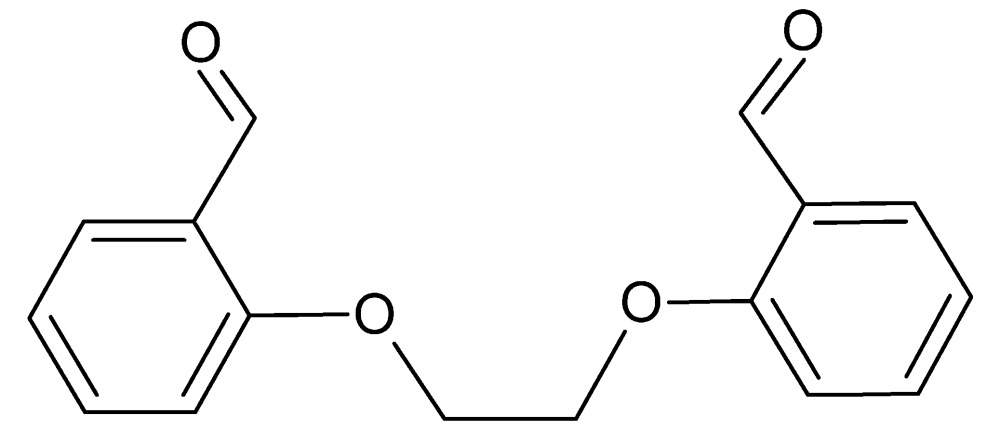


## Experimental

### 

#### Crystal data

C_16_H_14_O_4_
*M* *_r_* = 270.27
Triclinic, 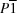

*a* = 7.7571 (1) Å
*b* = 8.3277 (1) Å
*c* = 11.2965 (1) Å
α = 82.283 (7)°
β = 75.839 (7)°
γ = 66.823 (6)°
*V* = 649.87 (4) Å^3^
*Z* = 2
Mo *K*α radiation
μ = 0.10 mm^−1^
*T* = 120 K
0.62 × 0.44 × 0.22 mm

#### Data collection

Rigaku R-AXIS conversion diffractometer
Absorption correction: multi-scan (*CrystalClear-SM Expert*; Rigaku, 2012 ▶) *T*_min_ = 0.878, *T*_max_ = 1.000
9715 measured reflections
2969 independent reflections
2862 reflections with *I* > 2σ(*I*)
*R*_int_ = 0.031

#### Refinement

*R*[*F* ^2^ > 2σ(*F* ^2^)] = 0.039
*wR*(*F* ^2^) = 0.101
*S* = 1.07
2969 reflections
182 parameters
H-atom parameters constrained
Δρ_max_ = 0.34 e Å^−3^
Δρ_min_ = −0.19 e Å^−3^

### 

Data collection: *CrystalClear-SM Expert* (Rigaku, 2012 ▶); cell refinement: *CrystalClear-SM Expert*; data reduction: *CrystalClear-SM Expert*; program(s) used to solve structure: *SHELXS97* (Sheldrick, 2008 ▶); program(s) used to refine structure: *SHELXL97* (Sheldrick, 2008 ▶); molecular graphics: *ORTEP-3 for Windows* (Farrugia, 2012 ▶); software used to prepare material for publication: *WinGX* (Farrugia, 2012 ▶) and *PLATON* (Spek, 2009 ▶).

## Supplementary Material

Crystal structure: contains datablock(s) global, I. DOI: 10.1107/S1600536813019156/bt6920sup1.cif

Structure factors: contains datablock(s) I. DOI: 10.1107/S1600536813019156/bt6920Isup2.hkl

Additional supplementary materials:  crystallographic information; 3D view; checkCIF report

## Figures and Tables

**Table 1:** Hydrogen-bond geometry (Å, °) *Cg*1 and *Cg*2 are the centroids of the C3–C8 and C10–C15 benzene rings, respectively.

*D*—H⋯*A*	*D*—H	H⋯*A*	*D*⋯*A*	*D*—H⋯*A*
C8—H8⋯O3^i^	0.95	2.44	3.2508 (17)	144
C2—H2*A*⋯*Cg*1^ii^	0.99	2.68	3.4220 (12)	132
C2—H2*B*⋯*Cg*2^iii^	0.99	2.70	3.5964 (14)	151

Symmetry codes: (i) 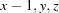; (ii) 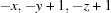; (iii) 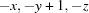.

**Fig. 1.**
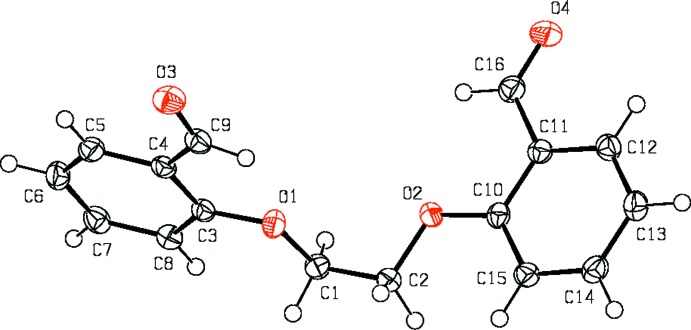
View of the molecular structure of the title compound with the atom numbering scheme. Displacement ellipsoids for non-H atoms are drawn at the 50% probability level.

**Fig. 2.**
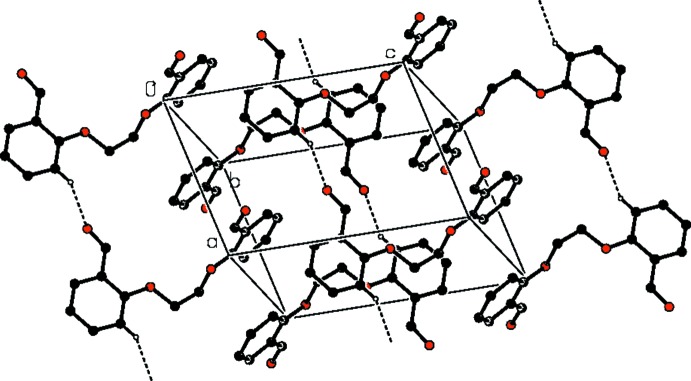
Partial packing diagram of the title compound showing hydrogen bonds as dashed lines. H atoms not involved in hydrogen bonds have been omitted for clarity.

## supplementary crystallographic information

### Comment

The synthesis of bis functionalized compounds has attracted the interest of chemists in chemical industry. Such compounds are considered as significant precursores for building blocks of vital molecules in different studies such as supramolecular chemistry and nanoscience (Holland *et al.*, 2007), bioactive bis heterocyclic compounds (Pedras *et al.*, 2010; Mabkhot *et al.*, 2012), and binucleating ligand designs (Gavrilova & Bosnich, 2004). In this concept the title compound has been synthesized among several derivatives of bis functionalized compounds as precursers for the synthesis of a series of macromolecular compounds.

The dihedral angle between the two benzene rings (C3–C8 and C10–C15) of the title compound (Fig. 1) is 75.14 (9)°. The torsion angle of the bridge O–C–C–O group is -76.50 (11)°. The C3–C4–C9–O3 and C10–C11–C16–O4 torsion angles of the two benzaldehyde groups are 175.10 (11) and -175.87 (13)°, respectively. Thus, they are almost coplanar with the rings to which they are attached. The bond lengths are normal (Allen *et al.*, 1987).

In the crystal, molecules are connected by C–H···O hydrogen bonds, generating infinite chains with the graph-set motif C(6) (Table 1, Fig. 2; Bernstein *et al.*, 1995) along the *a* axis. In addition, C–H···π interactions (Table 1) and π-π stacking interactions [*Cg*1···*Cg*1( - *x*, 2 - *y*, 1 - *z*) = 3.6957 (7) Å and *Cg*2···*Cg*2( 1 - *x*, 1 - *y*, 1 - *z*) = 3.6735 (8) Å; where *Cg*1 and *Cg*2 are the centroids of the C3–C8 and C10–C15 benzene rings, respectively] contribute to stabilize the crystal structure.

### Experimental

A solution of 1.22 g m (0.01 mol) salicyldehyde in hot ethanolic KOH (prepared by dissolving 560 mg (0.01 mol) KOH in 100 ml of absolute ethanol) was stirred until a clear solution was obtained, which was then evaporated under vacuum. The residue was dissolved in DMF (25 ml) and 940 mg (0.005 mol) of dibromoethane was added. The reaction mixture was refluxed for 5 minutes, during which KBr was separated out. The solvent was then removed in vacuo and the remaining solid was washed with water and crystallized from ethanol to give high quality crystals (*Mp*. 393 K) suitable for X-ray analysis in an good yield (84%).

### Refinement

All H atoms were found in a difference map, but placed geometrically with C—H = 0.95 Å (aromatic H) and 0.99 Å (methylene H) and were refined using a riding model with *U*_iso_(H) = 1.2*U*_eq_(C).

### Crystal data

**Table:**

C_16_H_14_O_4_	*Z* = 2
*M**_r_* = 270.27	*F*(000) = 284
Triclinic, *P*1	*D*_x_ = 1.381 Mg m^−^^3^
Hall symbol: -P 1	Mo *K*α radiation, λ = 0.71075 Å
*a* = 7.7571 (1) Å	Cell parameters from 4015 reflections
*b* = 8.3277 (1) Å	θ = 1.9–27.5°
*c* = 11.2965 (1) Å	µ = 0.10 mm^−^^1^
α = 82.283 (7)°	*T* = 120 K
β = 75.839 (7)°	Block, colourless
γ = 66.823 (6)°	0.62 × 0.44 × 0.22 mm
*V* = 649.87 (4) Å^3^	

### Data collection

**Table:**

Rigaku R-AXIS conversion diffractometer	2969 independent reflections
Radiation source: Sealed Tube	2862 reflections with *I* > 2σ(*I*)
Graphite Monochromator monochromator	*R*_int_ = 0.031
Detector resolution: 10.0000 pixels mm^-1^	θ_max_ = 27.5°, θ_min_ = 2.9°
profile data from ω–scans	*h* = −10→8
Absorption correction: multi-scan (*CrystalClear-SM Expert*; Rigaku, 2012)	*k* = −10→10
*T*_min_ = 0.878, *T*_max_ = 1.000	*l* = −14→14
9715 measured reflections	

### Refinement

**Table:**

Refinement on *F*^2^	Hydrogen site location: inferred from neighbouring sites
Least-squares matrix: full	H-atom parameters constrained
*R*[*F*^2^ > 2σ(*F*^2^)] = 0.039	W = 1/[Σ^2^(*F*O^2^) + (0.0397*P*)^2^ + 0.2051*P*] WHERE *P* = (*F*O^2^ + 2*F*C^2^)/3
*wR*(*F*^2^) = 0.101	(Δ/σ)_max_ < 0.001
*S* = 1.07	Δρ_max_ = 0.34 e Å^−^^3^
2969 reflections	Δρ_min_ = −0.19 e Å^−^^3^
182 parameters	Extinction correction: *SHELXL97* (Sheldrick, 2008), FC^*^=KFC[1+0.001XFC^2^Λ^3^/SIN(2Θ)]^-1/4^
0 restraints	Extinction coefficient: 0.039 (4)

### Special details

**Table:**

Geometry. Bond distances, angles *etc*. have been calculated using the rounded fractional coordinates. All su's are estimated from the variances of the (full) variance-covariance matrix. The cell e.s.d.'s are taken into account in the estimation of distances, angles and torsion angles
Refinement. Refinement on *F*^2^ for ALL reflections except those flagged by the user for potential systematic errors. Weighted *R*-factors *wR* and all goodnesses of fit *S* are based on *F*^2^, conventional *R*-factors *R* are based on *F*, with *F* set to zero for negative *F*^2^. The observed criterion of *F*^2^ > σ(*F*^2^) is used only for calculating -*R*-factor-obs *etc*. and is not relevant to the choice of reflections for refinement. *R*-factors based on *F*^2^ are statistically about twice as large as those based on *F*, and *R*-factors based on ALL data will be even larger.

### Fractional atomic coordinates and isotropic or equivalent isotropic displacement parameters (Å^2^)

**Table:**

	*x*	*y*	*z*	*U*_iso_*/*U*_eq_	
O1	0.02681 (11)	0.67683 (11)	0.39979 (7)	0.0234 (2)	
O2	0.09562 (12)	0.63138 (10)	0.13897 (7)	0.0229 (2)	
O3	0.42624 (12)	0.70766 (12)	0.54585 (8)	0.0310 (3)	
O4	0.29048 (14)	0.91784 (11)	−0.12808 (8)	0.0335 (3)	
C1	−0.10587 (16)	0.66169 (14)	0.33728 (10)	0.0222 (3)	
C2	0.00997 (16)	0.53887 (14)	0.23588 (10)	0.0221 (3)	
C3	−0.04430 (16)	0.76808 (13)	0.50371 (9)	0.0198 (3)	
C4	0.09320 (16)	0.78082 (14)	0.55953 (10)	0.0203 (3)	
C5	0.03276 (17)	0.87486 (14)	0.66511 (10)	0.0238 (3)	
C6	−0.16055 (18)	0.95186 (15)	0.71668 (10)	0.0264 (3)	
C7	−0.29501 (17)	0.93499 (15)	0.66234 (10)	0.0254 (3)	
C8	−0.23910 (16)	0.84489 (14)	0.55568 (10)	0.0225 (3)	
C9	0.29937 (16)	0.69362 (15)	0.50767 (10)	0.0235 (3)	
C10	0.20867 (15)	0.54157 (14)	0.03822 (10)	0.0201 (3)	
C11	0.28537 (16)	0.63720 (14)	−0.05755 (10)	0.0205 (3)	
C12	0.40808 (16)	0.55234 (15)	−0.16216 (10)	0.0237 (3)	
C13	0.45243 (17)	0.37632 (16)	−0.17322 (11)	0.0267 (3)	
C14	0.37194 (17)	0.28415 (15)	−0.07953 (11)	0.0255 (3)	
C15	0.25049 (17)	0.36520 (15)	0.02579 (10)	0.0228 (3)	
C16	0.23854 (17)	0.82470 (15)	−0.04713 (11)	0.0252 (3)	
H1A	−0.18070	0.77750	0.30370	0.0270*	
H1B	−0.19620	0.61520	0.39420	0.0270*	
H2A	0.11090	0.43660	0.26590	0.0270*	
H2B	−0.07370	0.49650	0.20600	0.0270*	
H5	0.12500	0.88620	0.70190	0.0290*	
H6	−0.20120	1.01580	0.78860	0.0320*	
H7	−0.42750	0.98600	0.69880	0.0300*	
H8	−0.33240	0.83580	0.51880	0.0270*	
H9	0.33550	0.62190	0.44020	0.0280*	
H12	0.46170	0.61600	−0.22640	0.0280*	
H13	0.53720	0.31880	−0.24420	0.0320*	
H14	0.40060	0.16380	−0.08780	0.0310*	
H15	0.19620	0.30080	0.08900	0.0270*	
H16	0.16270	0.87630	0.02780	0.0300*	

### Atomic displacement parameters (Å^2^)

**Table:**

	*U*^11^	*U*^22^	*U*^33^	*U*^12^	*U*^13^	*U*^23^
O1	0.0200 (4)	0.0302 (4)	0.0205 (4)	−0.0089 (3)	−0.0034 (3)	−0.0061 (3)
O2	0.0284 (4)	0.0212 (4)	0.0187 (4)	−0.0107 (3)	−0.0016 (3)	−0.0015 (3)
O3	0.0251 (5)	0.0358 (5)	0.0361 (5)	−0.0147 (4)	−0.0104 (4)	0.0025 (4)
O4	0.0444 (6)	0.0263 (4)	0.0336 (5)	−0.0190 (4)	−0.0086 (4)	0.0051 (4)
C1	0.0208 (5)	0.0257 (5)	0.0218 (5)	−0.0099 (4)	−0.0053 (4)	−0.0010 (4)
C2	0.0255 (6)	0.0231 (5)	0.0197 (5)	−0.0118 (4)	−0.0047 (4)	0.0009 (4)
C3	0.0227 (5)	0.0180 (5)	0.0180 (5)	−0.0084 (4)	−0.0031 (4)	0.0013 (4)
C4	0.0231 (5)	0.0192 (5)	0.0193 (5)	−0.0100 (4)	−0.0044 (4)	0.0035 (4)
C5	0.0310 (6)	0.0215 (5)	0.0222 (5)	−0.0123 (5)	−0.0090 (4)	0.0026 (4)
C6	0.0341 (6)	0.0218 (5)	0.0203 (5)	−0.0087 (5)	−0.0028 (5)	−0.0026 (4)
C7	0.0240 (6)	0.0220 (5)	0.0244 (5)	−0.0059 (4)	0.0002 (4)	−0.0003 (4)
C8	0.0215 (5)	0.0230 (5)	0.0228 (5)	−0.0090 (4)	−0.0043 (4)	0.0006 (4)
C9	0.0240 (6)	0.0250 (5)	0.0219 (5)	−0.0107 (4)	−0.0056 (4)	0.0035 (4)
C10	0.0199 (5)	0.0228 (5)	0.0190 (5)	−0.0077 (4)	−0.0068 (4)	−0.0013 (4)
C11	0.0210 (5)	0.0220 (5)	0.0210 (5)	−0.0093 (4)	−0.0077 (4)	0.0009 (4)
C12	0.0245 (6)	0.0262 (6)	0.0212 (5)	−0.0103 (4)	−0.0055 (4)	0.0005 (4)
C13	0.0284 (6)	0.0272 (6)	0.0217 (5)	−0.0079 (5)	−0.0029 (4)	−0.0045 (4)
C14	0.0299 (6)	0.0206 (5)	0.0264 (6)	−0.0082 (4)	−0.0079 (5)	−0.0030 (4)
C15	0.0264 (6)	0.0224 (5)	0.0219 (5)	−0.0111 (4)	−0.0071 (4)	0.0015 (4)
C16	0.0296 (6)	0.0234 (5)	0.0246 (5)	−0.0106 (5)	−0.0087 (5)	−0.0001 (4)

### Geometric parameters (Å, º)

**Table:**

O1—C1	1.4337 (16)	C12—C13	1.3834 (17)
O1—C3	1.3611 (13)	C13—C14	1.3910 (18)
O2—C2	1.4332 (14)	C14—C15	1.3876 (17)
O2—C10	1.3593 (13)	C1—H1A	0.9900
O3—C9	1.2166 (17)	C1—H1B	0.9900
O4—C16	1.2147 (15)	C2—H2A	0.9900
C1—C2	1.5011 (15)	C2—H2B	0.9900
C3—C4	1.4081 (18)	C5—H5	0.9500
C3—C8	1.3940 (18)	C6—H6	0.9500
C4—C5	1.3942 (15)	C7—H7	0.9500
C4—C9	1.4731 (18)	C8—H8	0.9500
C5—C6	1.3839 (19)	C9—H9	0.9500
C6—C7	1.392 (2)	C12—H12	0.9500
C7—C8	1.3906 (16)	C13—H13	0.9500
C10—C11	1.4090 (16)	C14—H14	0.9500
C10—C15	1.3929 (16)	C15—H15	0.9500
C11—C12	1.3951 (16)	C16—H16	0.9500
C11—C16	1.4717 (16)		
			
C1—O1—C3	118.45 (10)	C2—C1—H1A	110.00
C2—O2—C10	117.35 (8)	C2—C1—H1B	110.00
O1—C1—C2	107.09 (10)	H1A—C1—H1B	109.00
O2—C2—C1	108.23 (9)	O2—C2—H2A	110.00
O1—C3—C4	115.69 (11)	O2—C2—H2B	110.00
O1—C3—C8	124.17 (11)	C1—C2—H2A	110.00
C4—C3—C8	120.14 (10)	C1—C2—H2B	110.00
C3—C4—C5	119.49 (11)	H2A—C2—H2B	108.00
C3—C4—C9	120.26 (10)	C4—C5—H5	120.00
C5—C4—C9	120.24 (12)	C6—C5—H5	120.00
C4—C5—C6	120.50 (12)	C5—C6—H6	120.00
C5—C6—C7	119.52 (11)	C7—C6—H6	120.00
C6—C7—C8	121.24 (12)	C6—C7—H7	119.00
C3—C8—C7	119.08 (12)	C8—C7—H7	119.00
O3—C9—C4	124.12 (11)	C3—C8—H8	120.00
O2—C10—C11	116.23 (9)	C7—C8—H8	120.00
O2—C10—C15	123.99 (10)	O3—C9—H9	118.00
C11—C10—C15	119.79 (10)	C4—C9—H9	118.00
C10—C11—C12	119.48 (10)	C11—C12—H12	120.00
C10—C11—C16	120.45 (10)	C13—C12—H12	120.00
C12—C11—C16	120.07 (11)	C12—C13—H13	120.00
C11—C12—C13	120.59 (11)	C14—C13—H13	120.00
C12—C13—C14	119.48 (11)	C13—C14—H14	119.00
C13—C14—C15	121.06 (11)	C15—C14—H14	119.00
C10—C15—C14	119.57 (11)	C10—C15—H15	120.00
O4—C16—C11	124.30 (11)	C14—C15—H15	120.00
O1—C1—H1A	110.00	O4—C16—H16	118.00
O1—C1—H1B	110.00	C11—C16—H16	118.00
			
C3—O1—C1—C2	−173.57 (9)	C4—C5—C6—C7	−0.04 (16)
C1—O1—C3—C8	1.68 (15)	C5—C6—C7—C8	−1.24 (17)
C1—O1—C3—C4	−178.90 (9)	C6—C7—C8—C3	0.98 (17)
C10—O2—C2—C1	179.42 (10)	O2—C10—C11—C12	177.80 (11)
C2—O2—C10—C15	−2.20 (18)	O2—C10—C11—C16	−1.51 (18)
C2—O2—C10—C11	177.73 (11)	C15—C10—C11—C12	−2.27 (19)
O1—C1—C2—O2	−76.50 (11)	C15—C10—C11—C16	178.42 (12)
O1—C3—C4—C9	−1.99 (15)	O2—C10—C15—C14	−178.22 (12)
C8—C3—C4—C5	−1.78 (16)	C11—C10—C15—C14	1.9 (2)
O1—C3—C8—C7	179.93 (9)	C10—C11—C12—C13	1.0 (2)
C4—C3—C8—C7	0.54 (16)	C16—C11—C12—C13	−179.70 (13)
C8—C3—C4—C9	177.46 (10)	C10—C11—C16—O4	−175.87 (13)
O1—C3—C4—C5	178.78 (10)	C12—C11—C16—O4	4.8 (2)
C3—C4—C9—O3	175.10 (11)	C11—C12—C13—C14	0.7 (2)
C5—C4—C9—O3	−5.67 (18)	C12—C13—C14—C15	−1.1 (2)
C3—C4—C5—C6	1.53 (16)	C13—C14—C15—C10	−0.2 (2)
C9—C4—C5—C6	−177.71 (10)		

### Hydrogen-bond geometry (Å, º)

Cg1 and Cg2 are the centroids of the C3–C8 and C10–C15 benzene rings, respectively.

**Table:**

*D*—H···*A*	*D*—H	H···*A*	*D*···*A*	*D*—H···*A*
C8—H8···O3^i^	0.95	2.44	3.2508 (17)	144
C9—H9···O1	0.95	2.40	2.7412 (16)	101
C16—H16···O2	0.95	2.42	2.7561 (15)	101
C2—H2*A*···*Cg*1^ii^	0.99	2.68	3.4220 (12)	132
C2—H2*B*···*Cg*2^iii^	0.99	2.70	3.5964 (14)	151

Symmetry codes: (i) *x*−1, *y*, *z*; (ii) −*x*, −*y*+1, −*z*+1; (iii) −*x*, −*y*+1, −*z*.
